# Estimation of light utilisation and antioxidative protection in an alpine plant species (*Soldanella alpina* L.) during the leaf life cycle at high elevation

**DOI:** 10.1111/ppl.70045

**Published:** 2025-01-16

**Authors:** Peter Streb, Philippine Dubertrand, Gabriel Cornic, Kamel Soudani, Giovanni Finazzi

**Affiliations:** ^1^ Université Paris‐Saclay, Laboratoire Ecologie, Systématique et Evolution, Equipe Ecophysiologie Végétale, IDEEV Gif‐sur‐Yvette France; ^2^ Laboratoire de Physiologie Cellulaire et Végétale, UMR 5168, Centre National de la Recherche Scientifique (CNRS), Commissariat à L’énergie Atomique et aux Energies Alternatives (CEA), Université Grenoble Alpes, Institut National de Recherche Agronomique (INRA), Institut de Recherche en Sciences et Technologies pour le Vivant (iRTSV), CEA Grenoble, Grenoble cedex 9 France

## Abstract

Photosynthesis, electron transport to carbon assimilation, photorespiration and alternative electron transport, light absorption of the two photosystems, antioxidative protection and pigment contents were investigated in *S. alpina* leaves. *S. alpina* is an alpine snow‐bed plant which can be found with green leaves after snowmelt. At least 24% of the leaves were formed at the beginning of the vegetation period in the previous year and survived two consecutive vegetation periods under contrasting environmental conditions. In leaves still covered by snow (SNOW), the parameters of antioxidative protection and carbon assimilation were lower than in leaves from the previous vegetation period (NEW) or several weeks after snowmelt (OLD). Directly after snowmelt, antioxidative protection was strongly but transitionally increased. The senescence of leaves did not depend on antioxidative scavenging capacity. Lower carbon assimilation was not related to increases in alternative electron flow (ETR_alt_) in SNOW leaves. In the second vegetation period, light absorption by PSII decreases in favour of PSI in OLD leaves. This allows OLD leaves to keep the electron transport chain more oxidised and to support photorespiration with increased ATP synthesis by cyclic electron transport around PSI. This study describes how the leaves of a unique plant can cope with contrasting environmental conditions.

## 1 INTRODUCTION

**FIGURE 1:**
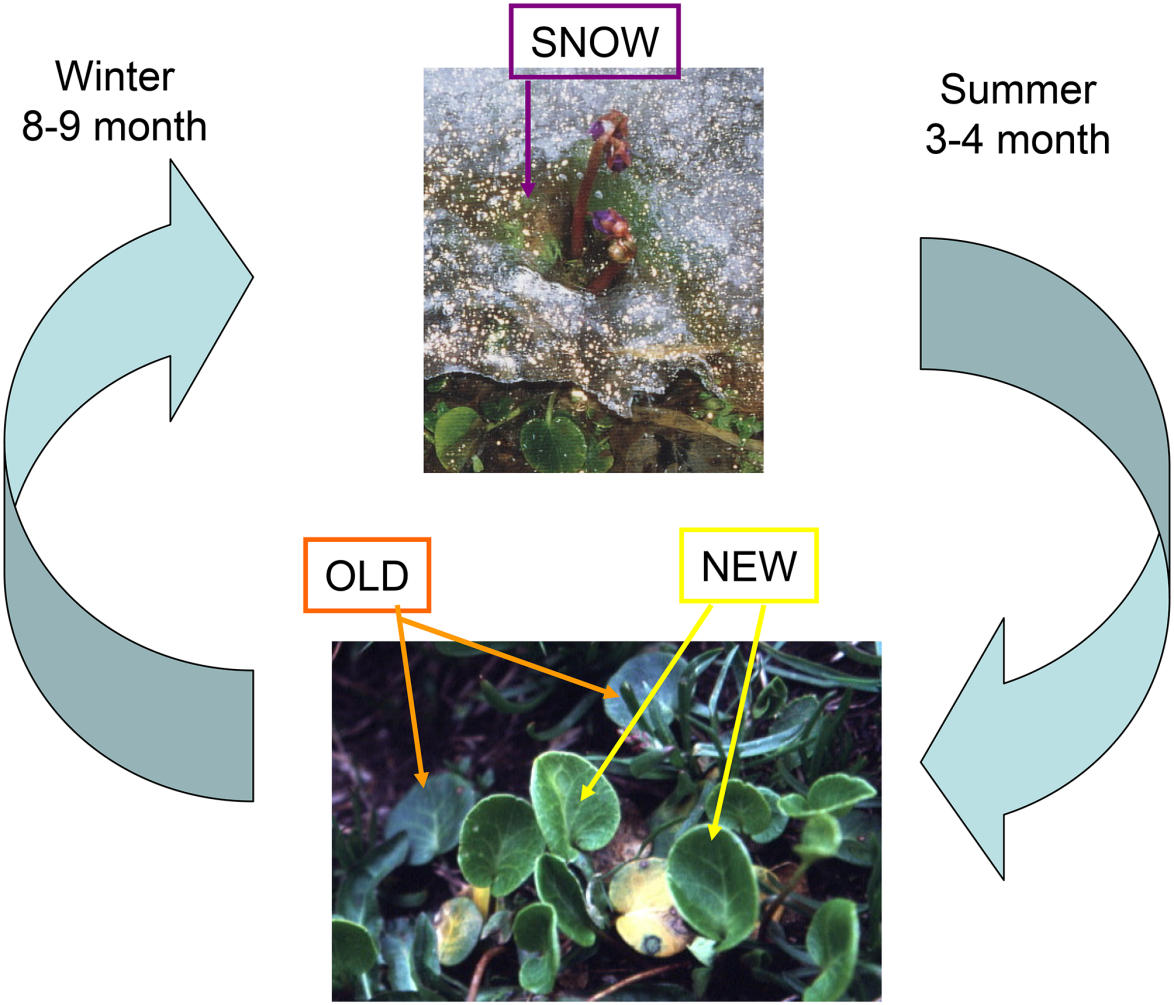
*Soldanella alpina* leaves used for the investigations at 2400 m altitude. At this altitude NEW leaves are formed in June (depending on the snow cover), survive the following winter under a high snowpack. Investigated SNOW leaves were still covered by snow in the following year and investigated also 3 and 10 days after snow‐melt. OLD intact leaves, as well as senescent leaves were investigated during the second vegetation period at least 3 weeks after snowmelt. The vegetation period is defined as the time between snowfall and snow‐melt.

*Soldanella alpina* L. leaves growing in the French Alps at 2400 m elevation were investigated (Figure 1). Similar to the well‐described *Soldanella pusilla* (Körner et al., 2019; Körner, 2021), green leaves of *S. alpina* survive the winter under snow (Streb & Cornic, 2012; Fernandez‐Marin, 2021). Rosette‐like leaves of *S. alpina* grow in a small distance above the ground. During the alpine summer, most leaves are overgrown by the surrounding vegetation. Therefore, *S. alpina* leaves experience contrasting environmental conditions during their life cycle. In spring, after snowmelt, the vegetation period starts and most newly formed leaves (NEW) develop in the shade or are shaded during the alpine summer. A shaded leaf history of the leaves can be identified by their low Dualex index, indicating low epidermal flavonoid contents compared to sun‐grown leaves (Laureau et al., 2015). In autumn, before snowfall, the shading vegetation gets senescent, and *S. alpina* leaves are exposed to direct sunlight. Some ability of shade‐grown *S. alpina* leaves to tolerate a sudden change to high light in the same vegetation period was previously demonstrated (Talhouët et al., 2020). During winter, leaves are covered by snow for eight to nine months, almost in darkness. The light intensity increases in spring, even when there is a low snow cover (Körner, 2021), and the leaf temperature rises rapidly directly after snowmelt (Streb & Cornic, 2012). During the following two to three weeks, leaves are exposed to full sunlight before they are again overgrown. Altogether, a single *S. alpina* leaf may experience shade, high light (up to 3000 μmol m^−2^ s^−1^ PFD), darkness and again high light and shade, while the leaf temperature can vary between 0 up to 40°C (Laureau et al., 2011) (Figure S1).

High photosynthetic light intensity induces photoinhibition and photooxidative damage in non‐acclimated plants, in particular, if combined with low or high temperatures (Streb & Cornic, 2012; Choudhury et al., 2017; Fernandez‐Marin et al., 2020). While high light intensity generates high photosynthetic electron transport, low and high temperatures decrease the activity of the Calvin‐Benson‐Bassham cycle, although for different reasons (Allakhverdiev et al., 2008; Streb & Cornic, 2012). As a consequence, electron transport can exceed electron consumption by carbon assimilation and photorespiration, the two major sinks of photosynthetically generated electrons (Cornic & Baker, 2012). PSII electron transport can be quantitatively estimated by chlorophyll fluorescence (Baker, 2008), while carbon assimilation and photorespiration can be estimated using photosynthetic modelling and measuring carbon assimilation in the presence and absence of oxygen (von Caemmerer, 2000; Streb et al., 2005; Laureau et al., 2013). Several reactions, like nitrogen and sulphur reduction (Scheibe & Dietz, 2012) and the maintenance of reduced thioredoxin (Cornic & Baker, 2012), consume photosynthetically generated electrons but belong to normal cellular metabolism. Thus, they are expected to be oxygen‐independent. Other reactions evacuate electrons to oxygen as a final acceptor, either directly from the plastoquinone pool to reduce oxygen to water with PTOX (Cornic & Baker, 2012, Alric & Johnson, 2017) or indirectly via the malate valve to feed mitochondrial (alternative) respiration (Scheibe, 2019). Electrons transferred at PSI to oxygen lead to the formation of the reactive oxygen species (ROS) superoxide and H_2_O_2_, which can be scavenged in the water–water cycle by antioxidants (Cornic & Baker, 2012; Alric & Johnson, 2017). Electrons which are not consumed increase the reduction state of the plastoquinone pool, as estimated by the ql parameter (Baker, 2008) and increase the risk of singlet oxygen formation and PSII damage (Krieger‐Liszkay et al., 2008).

While ROS formation can potentially mediate damage to PSII, particularly of the D1 protein, membranes and pigments, ROS are also involved in stress signalling, ameliorating the general stress response of leaves (Scheibe & Dietz, 2012; Choudhury et al., 2017).

*S. alpina* leaves are equipped with efficient protection mechanisms against variations in light and temperature. The capacity of NPQ and the activity of the xanthophyll cycle are high (Streb et al., 1998). Furthermore, a strong antioxidative protecting system, including high contents and activities of α‐tocopherol, superoxide dismutase, catalase, and exceptionally high ascorbate contents as well as high PTOX contents, are present in *S. alpina* leaves (Streb et al., 1998; Streb et al., 2003b; Laureau et al., 2011).

The present investigation compares photosynthesis, photorespiration and alternative electron flow, antioxidative protection and pigment contents in intact leaves during their life cycle in leaves formed in spring (NEW), in leaves still covered by snow (SNOW) after winter and in leaves several weeks after snowmelt (OLD). We estimated how many leaves from the previous vegetation period survive the winter till the middle of the second vegetation period. The aim was to investigate: (1) are SNOW leaves photosynthetically active (2) how much of the light absorbed by photosystems is used for photosynthetic electron transport (3) how much of these electrons are used for carbon assimilation, photorespiration and are distributed to alternative acceptors in NEW, SNOW and OLD leaves (4) are antioxidative protection mechanisms preserved during winter in SNOW leaves or rapidly induced after snowmelt. In summary, could any of the described parameters contribute to explaining the activity of *S. alpina* leaves during a second vegetation period after such drastic light and temperature changes?

## 2 MATERIALS AND METHODS

### 2.1 Plant material

All investigations were performed with *S. alpina* leaves growing at 2400 m altitude near the Galibier pass as described (Laureau et al., 2011) between the beginning of June and August in a period of more than 15 years. Except for 77 K‐spectra, which were measured in one year, all other experiments were repeated at least in two different years with different plants and leaves. Leaves were divided into three categories (Figure 1): (1) NEW: Leaves formed in the current year (2) SNOW: Leaves from the previous year, still covered with snow (3) OLD: Leaves from the previous year at least three weeks after snowmelt. Some experiments were done with leaves for 3 days (SNOW+3d) and 10 days (SNOW+10d) after snowmelt. In SNOW plants ascorbate contents were also measured in roots, leaf and flower stalks and flowers. Mean light and temperature data during the vegetation period at the level of *S. alpina* leaves at 2400 m elevation were previously published (Laureau et al., 2011), and general climatic data for the investigation periods can be obtained from https://www.romma.fr/station_24.php?id=237 for 2100 m elevation.

The temperature and light conditions of *S. alpina* leaves in melting snow are shown in Streb & Cornic (2012). Thermographic photos to show temperature extremes were taken from leaves covered by melting snow and several days snow free with a FLIR Camera (T620).

### 2.2 Measurements of leaf survival

In six subsequent years, 285 individual newly formed leaves were marked with tape at the leaf stalk at their natural growing site (approximately 50 leaves every year). In the following year, after snowmelt, the marked leaves were visibly divided into 4 categories: (1) green, healthy leaves, (2) leaves up to 50% senescent (visible chlorophyll loss), (3) leaves more than 50% senescent (4) dead leaves (no chlorophyll or completely dried). It should be noted that not all marked leaves could be found in the following year (Table 5). To investigate if OLD leaves survive the second vegetation period, 45 OLD leaves were marked after snowmelt at the beginning of July and visibly controlled for three weeks under natural conditions for senescence. To investigate whether NEW leaves were senescent during their first vegetation period, 100 NEW leaves were marked and controlled during 3 to 4 weeks of the current year.

### 2.3 Photosynthetic measurements

Plants from the growing site were transferred into pots and either measured directly after sampling or kept under the same conditions as sampled for up to 3 days. All measurements were done with leaves attached to the plant. Measurements were done with a Licor 6400XT, equipped with a fluorescent chamber. After attaching the leaf to the chamber, the chamber was further isolated with a plastic cover and placed in another closed transparent chamber to avoid any unwanted external gas diffusion (Laureau et al., 2013). The air was decarbonated with soda lime and subsequently passed through a Licor 610 to set the humidity of the air. The leaf temperature was 25°C, and humidity was chosen to reach a vapour pressure deficit of approximately −1 kPa in the leaf. For every single event of the *A*_*N*_/light curve and of the *A*_*N*_/CO_2_ curve, carbon assimilation, maximum fluorescence (Fm′, 8600 μmol m^−2^ s^−1^ PFD, 1.2 s flash) Ft (fluorescence emission in light) and Fo′ (minimum fluorescence emission in light, measured after 6 s far‐red light), was measured. A list of abbreviations used is given in Table S2.

In general, the following protocol was applied, always after stabilising net carbon assimilation (*A*_*N*_) or dark respiration (*R*_*N*_):

- Dark acclimation for at least 30 min, measuring *R*_*N*_ and Fo.
- A saturating light pulse (8600 μmol m^−2^ s^−1^ PFD, 1.2 s) for measuring Fm.
- Light acclimation at 750 μmol m^−2^ s^−1^ PFD until *A*_*N*_ and stomatal conductance (*gs*) stabilized.
- *A*_*N*_/light curve at 25°C and 400 ppm CO_2_ (*C*_*A*_) and atmospheric oxygen (starting at 750 μmol m^−2^ s^−1^ PFD, increasing to 2000 μmol m^−2^ s^−1^ PFD and subsequently decreasing to 50 μmol m^−2^ s^−1^ PFD).
- *A*_*N*_/CO_2_ curve at 25°C and 750 μmol m^−2^ s^−1^ PFD (starting at 400 ppm *C*_*A*_, decreasing to 50 ppm *C*_*A*_ and increasing to 2000 ppm *C*_*A*_.
- Repetition of the *A*_*N*_/CO_2_ and *A*_*N*_/light curves in the absence of oxygen, replacing ambient air with nitrogen.
- Return to atmospheric conditions and measurements of respiration in light (*R*_*L*_) and C* in the absence of fluorescence, starting at 100, 200, 400 and 600 μmol m^−2^ s^−1^ PFD with steps of 10 ppm *C*_*A*_ from 50 to 120 ppm *C*_*A*_.

### 2.4 Calculations

Calculations of **ɸ**PSII, NPQ and ql were described previously (Baker, 2008)

$$ɸ\text{PSII}=\frac{\mathrm{Fm}\prime -\text{Ft}}{\mathrm{Fm}\prime }$$

$$\mathrm{NPQ}=\frac{\mathrm{Fm}-\mathrm{Fm}\prime }{\mathrm{Fm}\prime }$$

$$\mathrm{ql}=\frac{\mathrm{Fm}\prime -\text{Ft}}{\mathrm{Fm}\prime -\mathrm{Fo}\prime }\times \frac{\mathrm{Fo}\prime }{\text{Ft}}$$

Respiration in light (*R*_*L*_) and C* were determined as described previously with the Laisk method modified by mathematical calculation as described by Walker et al. (2016).

The apparent specificity factor of Rubisco was calculated by Laureau et al. (2013):

$$S_{\text{appci}}\mathrm{CO}=\frac{21000\times 0.028}{2\times C^{*}\times 0.759}$$

Where 21000 (μmol mol^−1^) is the oxygen molar ratio in air and 0.028 and 0.759 are the solubility factors of oxygen and CO_2_ at 25°C. The specificity factors and C* are shown in Table 1.

**TABLE 1:** C* and apparent specificity factors of RUBISCO of **NEW**, **SNOW** and **OLD** leaves as determined by gas exchange using *C*_*I*_.

	NEW	SNOW	OLD
C*	37.2	38.1	40.2
*S*_*appci*_	105	102	97

All values of C* are in the range of Γ* published by Evans and Loreto (2000), either *in vitro* or with gas exchange. According to von Caemmerer (2000), the determination of C* compared to Γ* results in lower values and higher specificity factors for RUBISCO. In accordance, *S*_*appci*_ is in the range of RUBISCO specificity factors measured in vitro but at the highest values (von Caemmerer, 2000; Galmes et al., 2005). Since C* effects calculated electron transport to carboxylation (ETRC) and oxygenation (ETRO), we tested a possible variation of C* in OLD leaves (40.2 ± 5). The total electron transport (ETRC + ETRO) varied at the highest PFD by less than ±5 e^−^ μmol m^−2^ s^−1^.

Knowing the specificity factor of Rubisco allowed the calculation of *V*_*c*_ */V*_*o*_, using *C*_*I*_ (μmol mol^−1^) and the solubility factors for oxygen and CO_2_ (Laureau et al., 2013):

VcVo=0.759×CI21000×0.028×SappciCO=φ

*V*_*c*_ and *V*_*o*_ were then calculated (Laureau et al., 2013):

$$V_{c}=\frac{\left(A_{N}+R_{L}\right)}{1-\left(\frac{0.5}{\varphi }\right)}\;\text{and}\;V_{o}=\frac{V_{C}}{\varphi }$$

The electron transport to carboxylation ETRC was calculated, assuming that 4 electrons are needed for carbon assimilation (ETRC = 4 × *V*_*C*_) (Laureau et al., 2013) and the electron transport to photorespiration by assuming that 6 electrons are needed in the photorespiration cycle including 2 electrons for the reassimilation of liberated CO_2_ (ETRO = 6 × *V*_*O*_). This calculation differs from previously published results where CO_2_ reassimilation was not taken into account (Laureau et al., 2013), leading to higher alternative electron flow (ETR_alt_). The total electron transport to carbon assimilation and photorespiration is then given by ETRC+ETRO. The results were compared to the calculation of electron transport to carboxylation and photorespiration (*J*_*A*_) as given in von Caemmerer (2000) using Γ* = C*:

$$J_{A}=\frac{\left(A_{N}+R_{L}\right)\left(4\times C_{i}+8\times \Gamma^{*}\right)}{\left(C_{i}-\Gamma^{*}\right)}$$

Results of the comparison are shown in Figure S3 (supporting information) for one of the *A*_*N*_/light curves. Both calculations were not identical, but deviations were small (up to 2 e^−^ μmol m^−2^ s^−1^). The advantage of our approach was to differentiate electron transport from carbon assimilation and photorespiration and to take the reassimilation of photorespiratory CO_2_ into account or not.

The total electron transport at PSII (*Jt*) was calculated using two approaches.

*Jt* was calculated as described by Krall & Edwards (1992), suggesting that both photosystems absorb 50% of the incident light. The light transmission and reflection of *S. alpina* leaves were measured between 350 and 2500 nm with an integrating sphere coupled with a spectrophotometer (ASD Integrating Sphere and ASD FieldSpec 4 Hi‐Res, ASD products, Malvern Panalytical Ltd., UK). The light absorption was calculated for the wavelength range of the Licor light source. The light absorption of all leaf types in this part of the spectrum is shown in Table 2.

Jt=0.5×ɸPSII×PFD×Abs

**TABLE 2:** Light absorption of **NEW** (n = 5), **SNOW** (n = 10) and **OLD** (n = 5) leaves of *S. alpina* in the blue and red emission range of the Licor LED light source. Number of individual measured leaves (n).

Leaf absorption	NEW	SNOW	OLD
blue (10%)	0.94 ± 0.003	0.96 ± 0.009	0.93 ± 0.009
red (90%)	0.89 ± 0.011	0.90 ± 0.01	0.92 ± 0.004

PSII electron transport was also recalculated (Jt*) as described previously (Laureau et al., 2013) with modifications as described and discussed in supporting information S3 (Calculation of PSII electron transport). In brief, ɸPSII was correlated to ɸCO_2_ in the absence of oxygen. Contrary to previous publications (Genty et al., 1990; Ghashghaie & Cornic, 1994; Streb et al., 2005; Laureau et al., 2013), a non‐linear polynomial correlation was found to match best between ɸPSII and ɸCO_2_ (supporting information S4). For the calculation of the electron transport under atmospheric conditions (Jt*), the polynomial correlations shown in Table 3 were used.

**TABLE 3:** Polynomial correlation between ɸCO_2_ and ɸPSII in the absence of oxygen, separately measured for **NEW**, **SNOW** and **OLD** leaves. This calculation was used to recalculate *Jt* ^*^ under atmospheric conditions.

NEW	SNOW	OLD
ɸCO_2_* = 0.002 + (0.111 ɸPSII) + (0.036 ɸPSII^2^)	ɸCO_2_* = 0.002 + (0.076 ɸPSII) + (0.072 ɸPSII^2^)	ɸCO_2_* = 0.001 + (0.1 ɸPSII) + (0.038 ɸPSII^2^)

The electron transport was then recalculated:

$${\mathrm{Jt}}^{*}={{\mathrm{ɸCO}}_{2}}^{*}\times 4\times {\mathrm{PFD}}_{\mathrm{abs}}$$

To assess alternative electron flow (ETR_alt_) not related to carbon assimilation and photorespiration;

$${\mathrm{ETR}}_{\mathrm{alt}}={\mathrm{Jt}}^{*}–\left(\text{ETRC}+\text{ETRO}\right)$$

### 2.5 Fluorescence spectra at 77 K

*S. alpina* leaves sampled at their natural growing site were immediately frozen in liquid nitrogen and rapidly transferred to the laboratory, where they were ground with liquid nitrogen and ‘diluted’ with quartz sand. To avoid auto absorption of fluorescence, spectra were recorded with a homemade fluorimeter. Excitation and emission were at 450 nm and between 650 and 800 nm, respectively. Spectra were normalized to emission at 734 nm, the maximum fluorescence emission of PSI.

### 2.6 Metabolite and pigment content and enzyme activity

The plant material was either frozen in liquid nitrogen at the growing site or transported on ice to the laboratory and extracted immediately. Frozen material was extracted in liquid nitrogen and thawed in the presence of the extraction medium.

Enzyme activities were extracted in 50 mM potassium‐phosphate buffer pH 7.5 at 4°C, centrifuged, and the supernatant was used for measurements at 25°C. Catalase (EC1.11.1.6), glycolate oxidase (EC 1.1.3.1), glutathione reductase (EC 1.6.4.2) and cytoplasmic ascorbate peroxidase (EC 1.11.1.11) were measured as described in Laureau et al. (2011).

Ascorbic acid and glutathione were measured enzymatically and extracted and determined as described previously (Laureau et al., 2011).

Graphics and tables show mean values and standard errors. The number of independent repetitions (n) is indicated in the figures and tables.

Antioxidant contents, enzyme activities and pigment contents of the different leaf types were tested pairwise for significant differences at the *p* < 0.05 level (*t*‐test, Sharpiro‐Wilk). These results are shown in Table S5. Gas exchange measurements were tested by one‐way ANOVA (Holm‐Sidak method), and significant differences between OLD and SNOW leaves compared to NEW leaves were marked with asterisks in the graphics. For statistical analysis, the Sigma Plot program was used.

## 3 RESULTS

### 3.1 Photosynthetic parameters

**FIGURE 2:**
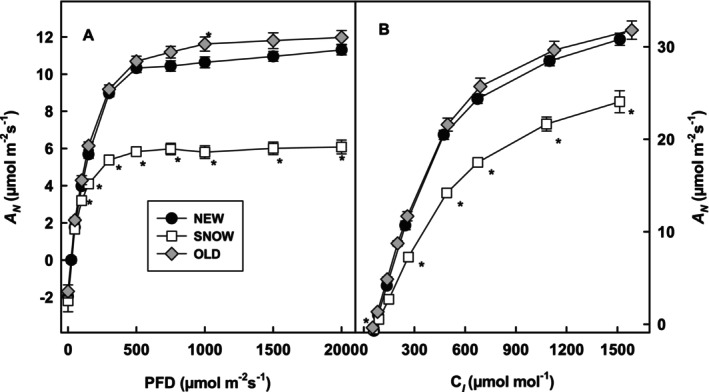
Photosynthetic net assimilation (*A*_*N*_) in μmol m^−2^ s^−1^ as function of photon flux density (A) and as a function of *C*_*I*_ (B) in NEW (*n* ≥ 9), SNOW (*n* ≥ 5) and OLD (*n* ≥ 7) leaves. Significant difference at the *p* < 0.05 level compared to NEW leaves is shown with asterisks.

SNOW leaves were clearly photosynthetic active. However, *A*_*N*_ was significantly lowest in SNOW leaves and slightly highest in OLD leaves, irrespective of whether it was measured as a function of PFD or as a function of *C*_*I*_. *A*_*N*_ saturated at 500 μmol m^−2^ s^−1^ PFD in SNOW leaves and at around 1000 μmol m^−2^ s^−1^ PFD in NEW and OLD leaves (Figure 2A). *A*_*N*_ did not saturate at the highest *C*_*I*_ in all leaf types (Figure 2B). Nevertheless, photorespiration was inhibited in all leaf types at the highest *C*_*I*_, since *A*_*N*_ was the same in ambient atmosphere and in an atmosphere with low oxygen (not shown). *A*_*N*_ and light saturation in NEW and OLD leaves at 2400 m elevation was higher than previously published for NEW sun leaves at 2100 m elevation (Talhouët et al., 2019). Interestingly, the light saturation of *A*_*N*_ corresponded well to the mean day PFD at the altitude of the measurement (650 μmol m^−2^ s^−1^ PFD at 2100 m and 950 μmol m^−2^ s^−1^ PFD at 2400 m) (Talhouët et al., 2019 and Figure 2A).

**FIGURE 3:**
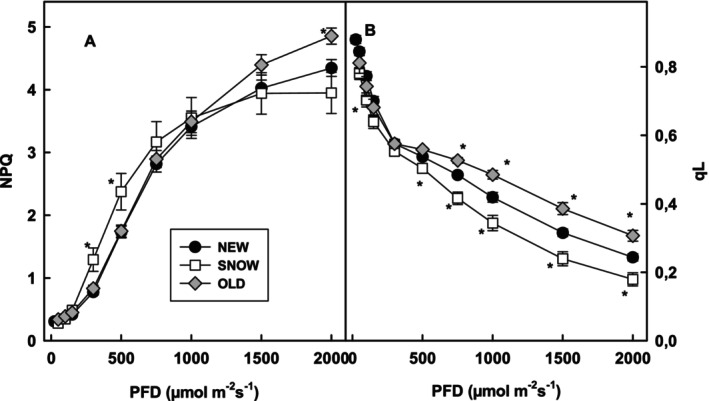
Non‐photochemical quenching (A) (NPQ) and qL (B) as function of photon flux density in NEW (n ≥ 9), SNOW (n ≥ 5) and OLD (n ≥ 7) leaves. Significant difference at the p < 0.05 level compared to NEW leaves is shown with asterisks.

Energy emission as heat, as estimated by NPQ, was highest in OLD and lowest in SNOW leaves at the highest PFD. At low PFD up to 750 μmol m^−2^ s^−1^ PFD, NPQ was highest in SNOW leaves (Figure 3A). The relative oxidation state of PSII (ql) was lowest in SNOW leaves. In NEW leaves, ql was higher than in OLD leaves, up to 300 μmol m^−2^ s^−1^ PFD. At increasing PFDs, ql was highest in OLD leaves (Figure 3B).

### 3.2 Electron transport

**FIGURE 4:**
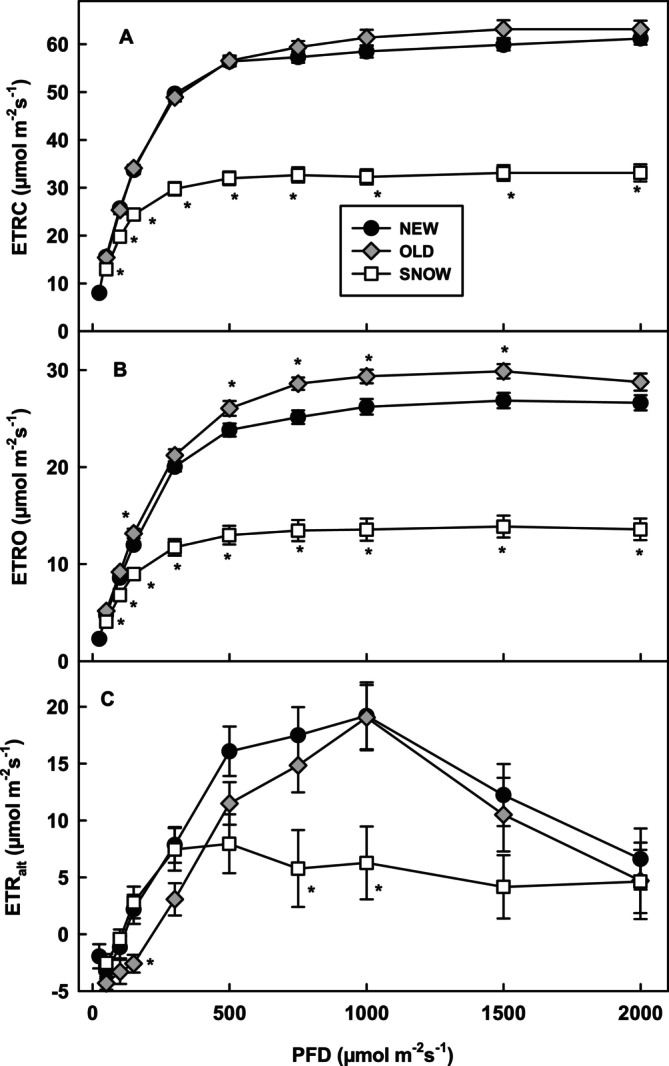
Calculated electron transport to the carboxylation (ETRC) and oxygenation (ETRO) reaction of Rubisco and alternative electron flow (ETR_alt_ = Jt*‐ (ETRC+ETRO) as function of photon flux density in NEW (n ≥ 9), SNOW (n ≥ 5) and OLD (*n* ≥ 7) leaves. Significant difference at the p < 0.05 level compared to NEW leaves is shown with asterisks.

**FIGURE 5:**
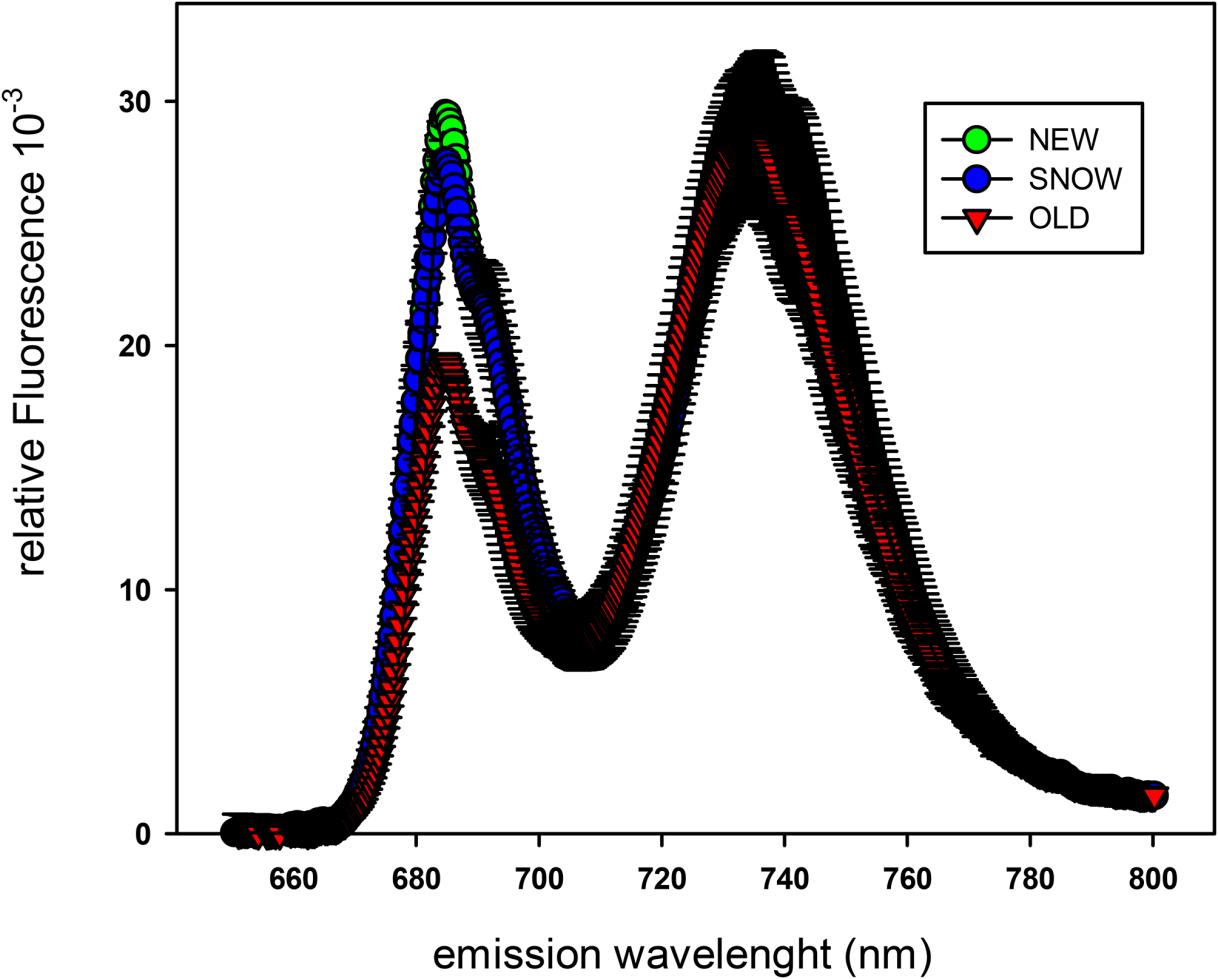
77 K chlorophyll fluorescence emission spectra of NEW, SNOW and OLD leaves of *S. alpina* (*n* = 4). Chlorophyll fluorescence was excited with blue light (450 nm) and emission spectra were recorded between 650 and 800 nm. The spectra were calibrated to the maximum fluorescence emission of PSI at 734 nm. The corresponding Fv/Fm ratio was: NEW = 0.79 ± 0.01; SNOW = 0.74 ± 0.02, OLD = 0.81 ± 0.01.

As expected, most electrons passing PSII were distributed to carbon assimilation in all leaf types (ETRC, Figure 4A). Surprisingly, relative to total PSII electron flux (Jt*)>, SNOW leaves used the highest percentage of transported electrons for carbon assimilation at 1000 μmol m^−2^ s^−1^ PFD (Table 4). Electron transport to photorespiration (ETRO) was highest in OLD leaves (Figure 4B), but similar in all leaves types when calculated as percent of Jt* (Table 4). Leaf types mostly differed in electron transport to alternative acceptors. ETR_alt_ was highest in NEW and lowest in SNOW leaves. The appearance of a negative ETR_alt_ at low PFD is discussed in supporting information S4. At PFDs up to light saturation, ETR_alt_ was delayed in OLD compared to NEW leaves, probably because light absorption by PSII was lowest (Figure 5). ETR_alt_ increased in all leaf types up to light saturation and then rested stable (SNOW) or decreased (NEW and OLD).

**TABLE 4:** Calculated electron transport at PSII Jt* or calculated electron transport according to Krall & Edwards (1992) *Jt*, electron transport to the carboxylation (ETRC) and the oxygenation (ETRO) reaction of Rubisco, alternative electron flow (ETR_alt_) and comparison of alternative electron flow with two other approaches: electron flow at PSII in the absence of oxygen and ETRO was substracted from electron flow under atmospheric conditions (Jt*_atm_‐ETRO‐Jt*_N2_) and electron flow to carboxylation and oxygenation was calculated according to von Caemmerer (2000) (Jt* ‐ *J*_*A*_) in **NEW**, **SNOW** and **OLD** leaves of *S. alpina* at 1000 μmol m^−2^ s^−1^ PFD. The percentage of electrons used for ETRC, ETRO and ETR_alt_ is indicated.

1000 μmol m^−2^ s^−1^ PFD	NEW	SNOW	OLD
Jt* μmol m^−2^ s^−1^	103.4 ± 2.5 (100%)	52.1 ± 4.6 (100%)	109.7 ± 3.0 (100%)
*Jt* (Krall & Edwards, 1992) μmol m^−2^ s^−1^	100.8 ± 2.5	64.6 ± 6.0	120.5 ± 2.7
ETRC: μmol m^−2^ s^−1^	58.6 ± 1.7 (57%)	32.3 ± 1.6 (62%)	61.4 ± 1.7 (56%)
ETRO μmol m^−2^ s^−1^	26.2 ± 0.8 (25%)	13.6 ± 1.4 (26%)	29.4 ± 0.7 (27%)
Jt*_atm_‐ETRO‐Jt*_N2_ μmol m^−2^ s^−1^	22.3 ± 4.5	7.0 ± 4.7	21.4 ± 2.9
ETR_alt_ μmol m^−2^ s^−1^	19.2 ± 2.9 (18%)	6.3 ± 3.2 (12%)	19.0 ± 2.9 (17%)
Jt* ‐*J*_*A*_ μmol m^−2^ s^−1^ (von Caemmerer, 2000)	17.4 ± 3.0	5.4 ± 3.2	17.0 ± 2.9

**TABLE 5:** Survival of *S. alpina* leaves during winter. Leaves were marked at the beginning of July every year and markers were counted one year later. Markers found were visibly divided into green healthy leaves, leaves with up to 50% senescent, leaves with higher senescence, dead leaves and markers without leaves.

green healthy	up to 50% senescent	senescent	Dead	markers without leaves
24 ± 5.8%	9 ± 1%	7 ± 3.3%	9 ± 3.3%	51 ± 7%

In order to verify the calculation of Jt* and ETR_alt_, comparisons were made to other approaches at 1000 μmol m^−2^ s^−1^ PFD (Table 4). Calculation of *Jt* according to Krall & Edwards (1992) was higher in SNOW and OLD leaves compared to Jt*, resulting in a higher ETR_alt_. In NEW leaves, results were similar (Table 4). Alternative electron flow was further estimated as the difference between Jt* under atmospheric conditions and Jt* in the absence of oxygen and ETRO (Jt*_atm_‐ETRO‐Jt*_N2_). This calculation is mainly based on electron transport measured under two atmospheric conditions and calculated photorespiration. Alternative electron flow calculated this way was slightly higher than ETR_alt_. Furthermore, alternative electron flow was calculated using *J*_*A*_ (von Caemmerer 2000), which resulted in a slightly lower alternative electron flow than ETR_alt_. All values calculated with different methods were within the standard error of our calculation of ETR_alt_ (Table 4).

### 3.3 77 K fluorescence emission spectra

The distribution of absorbed light energy between PSII and PSI was estimated with fluorescence emission spectra at 77 K (Figure 5). The spectra were calibrated at the maximum PSI emission peak. Fluorescence emission of PSII (685 nm) decreased slightly during winter in SNOW compared to NEW leaves. In OLD leaves, PSII was markedly lower than in NEW leaves. Obviously, light absorption of PSII decreased progressively in favour of PSI from the first to the second season. The changing light absorption to both photosystems explains, at least partially, the different correlations between ɸPSII and ɸCO_2_, as shown in Table 2.

### 3.4 Antioxidant content and enzyme activity

**FIGURE 6:**
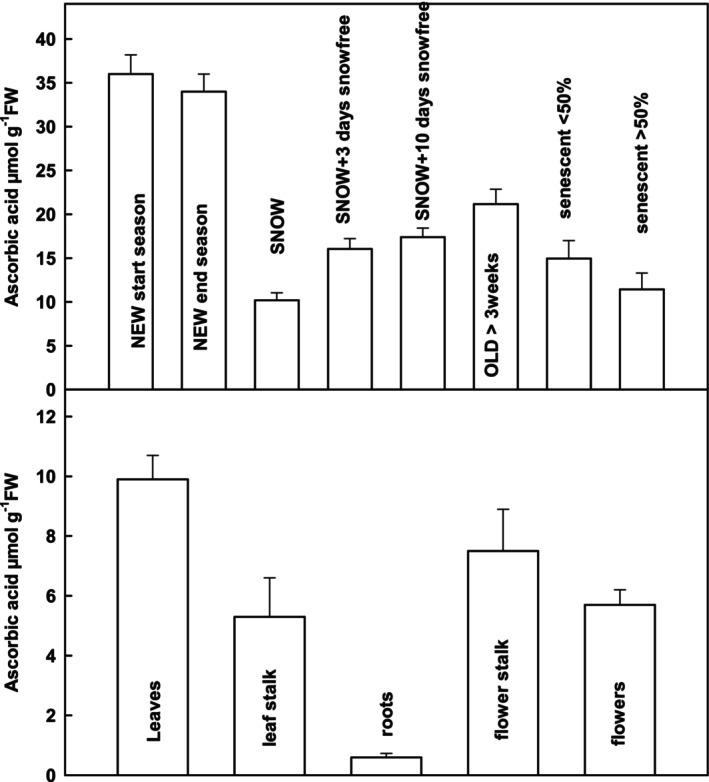
A) Reduced ascorbic acid content in *S. alpina* leaves during their life cycle. NEW leaves were measured at the beginning (*n* = 11) of the growing season and at the end (*n* = 22). SNOW (*n* = 34) leaves were collected under snow before melting in spring. SNOW+3 days (*n* = 23) and SNOW+10 days (*n* = 25) leaves were collected 3 or 10 days after snow‐melt. OLD (*n* = 19) leaves were collected several weeks after snowmelt. Senescent leaves were divided into leaves with visibly less (<) (*n* = 15) or more (>) (*n* = 17) than 50% senescence. Significant differences between the leaves are shown in supporting information S5. B) Distribution of reduced ascorbate between leaves (n = 34), the leaf stalk (*n* = 9), roots, (n = 9) the flower stalk (*n* = 5) and flowers (*n* = 16) in SNOW plants of *S. alpina*.

High antioxidant contents and high enzyme activities were not preserved in SNOW leaves. High ascorbate contents were confirmed in NEW leaves from the beginning to the end of the vegetation period (Figure 6A). However, ascorbate contents decreased significantly in winter under snow. In SNOW plants, most of the reduced ascorbate was still found in leaves, but important contents also in leaf stalks, flower stalks and flowers (Figure 6B). After snowmelt, ascorbate contents recovered immediately in leaves but remained significantly lower in OLD compared to NEW leaves. During leaf senescence, ascorbate contents of leaves decreased but remained as high as in SNOW leaves, even in strongly senescent leaves (Figure 6A).

**FIGURE 7:**
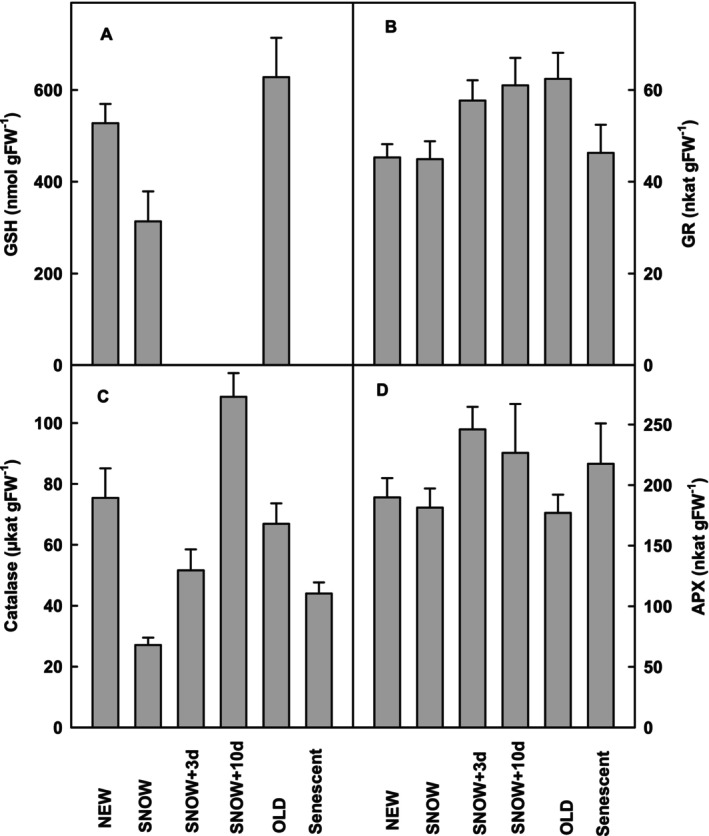
A) Reduced glutathione (GSH) content (*n* ≥ 3), B) glutathione reductase (GR) activity (n ≥ 7), C) catalase activity (n ≥ 7) and D) ascorbate peroxidase (APX) activity (*n* ≥ 6) in *S. alpina* leaves during their life cycle. NEW leaves were measured during the growing season. SNOW leaves were collected under snow before melting in spring. SNOW+3 days and SNOW+10 days were leaves collected 3 or 10 days after snowmelt. OLD leaves were collected several weeks after snow‐melt. Senescent leaves were visible senescent. Significant differences between the leaves are shown in supporting information S5.

Other parameters were investigated in less detail (Figure 7). The reduced glutathione content decreased in SNOW leaves but accumulated to higher values in OLD than in NEW leaves (Figure 7A). Similarly, glutathione reductase activity was higher in OLD than in NEW leaves, and the activity increased rapidly after snowmelt. Interestingly, glutathione reductase activity was similar in NEW, SNOW and senescent leaves (Figure 7B). Catalase activity was similar in NEW and OLD leaves but strongly decreased during winter in SNOW leaves and increased even more pronounced than other measured parameters 10 days after snowmelt (Figure 7C). Ascorbate peroxidase activity was similar in NEW, SNOW and OLD leaves but increase shortly after snow‐melt and in senescent leaves (Figure 7D).

### 3.5 Chlorophyll and carotenoid contents

**FIGURE 8:**
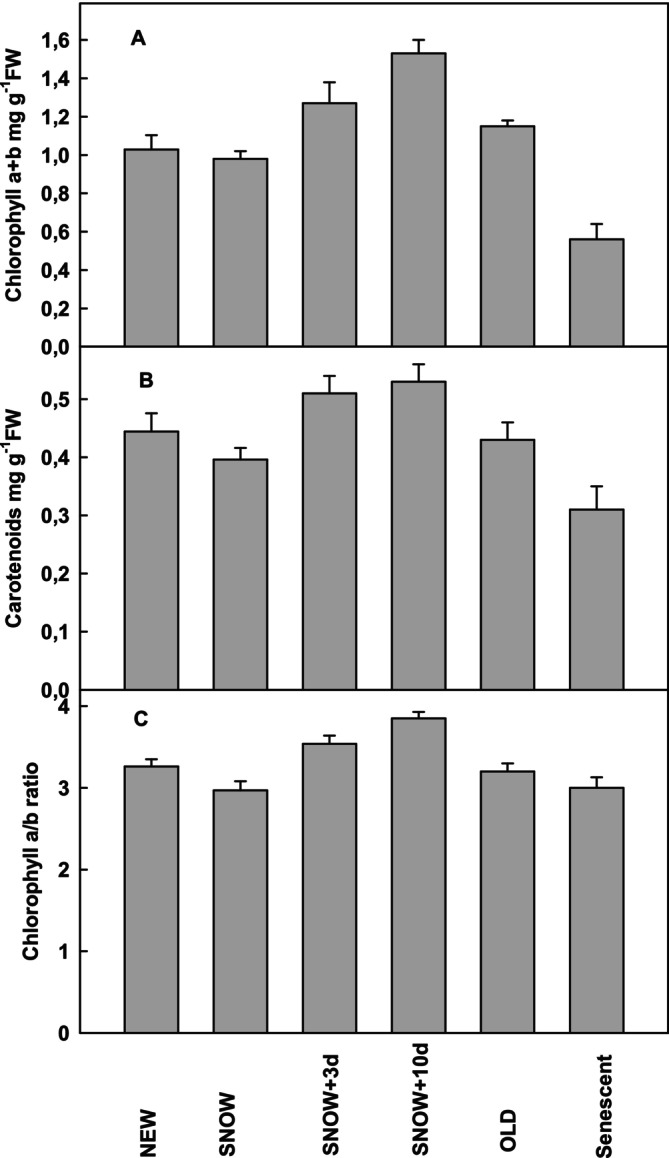
A) RT Chlorophyll a + b content, B) carotenoid content and C) chlorophyll a/b ratio in NEW (*n* = 10), SNOW (*n* = 27), SNOW+3 days snow‐free (n = 10), SNOW+10 days snow‐free (n = 10), OLD (n = 15) and senescent leaves (*n* = 4) of *S. alpina*. Significant differences between the leaves are shown in supporting information S5.

The total chlorophyll (Chla+b) and the carotenoid content were similar in NEW, SNOW and OLD leaves but decreased in senescent leaves (Figure 8A). The decrease of Chla+b content in senescent leaves was more pronounced than the decrease in carotenoids (Figure 8B). The Chla/b ratio was slightly lower in SNOW and senescent leaves than in NEW and OLD leaves, suggesting preferential degradation of reaction centres during winter and senescence. However, within 3 to 10 days after snowmelt, the total chlorophyll content, the Chl *a*/*b* ratio, and the carotenoid content increased markedly before decreasing several weeks after snowmelt in OLD leaves.

### 3.6 Leaf survival

In order to investigate how many leaves from the first year survive a second vegetation period, leaves were marked with tapes for five consecutive years. Of 285 markers, 98% were found in the following year. However, 51% of the markers were not attached to a leaf. It is impossible to decide whether the corresponding leaves were dead or the markers detached for other reasons. At least 24% of marked leaves were healthy and green, and 16% of leaves were strongly senescent or dead (Table 5). In order to investigate senescence in the present vegetation period, 50 OLD leaves and 100 NEW leaves were marked after snow‐melt/leaf formation in two subsequent years. 68% of OLD leaves and 90% of NEW leaves remained green till the beginning of August (not shown). We further found one two‐year‐old green, healthy leaf, and two leaves started getting senescent after a second winter period. The low yield of leaves surviving two winter periods may be explained by the fact that most marked leaves were taken from their growing site for measurements. It can be concluded that at least some leaves can survive two winters with snow cover and are even active during a third vegetation period.

## 4 DISCUSSION

The main outcome of this work is the functional characterisation of *S. alpina* leaves during their lifecycle: NEW leaves formed in the current year, SNOW leaves still covered by snow and OLD leaves during their second vegetation period. At least 24% of leaves formed in the first year are still photosynthetically active during a second vegetation period, and some individuals survive even a third period, with extreme light and temperature changes within a few days.

Photosynthetic activity in overwintering green plants has so far been characterized in conifer needles and wheat leaves (Savitch et al., 2002) but not in herbs still covered by snow. Similar to conifer needles, photosynthetic activity in SNOW leaves is downregulated, but *J*_t_* and ETRC are still higher than 50% compared to NEW and OLD leaves. In conifer needles shifted to 25°C, net carbon assimilation was reduced to 1/3, and PSII efficiency in light to 25% of those in non‐acclimated needles (Savitch et al., 2002).

Besides low temperatures during snowmelt, leaf temperature can rise up to 40°C in the sun a few days later (Figure S1). Since PFDs at this elevation may be as high as 3000 μmol m^−2^ s^−1^ (Streb & Cornic, 2012), light saturation of *A*_*N*_ can be exceeded by a factor 3 in NEW and OLD leaves and by a factor of 6 in SNOW leaves as measured at 25°C (Figure 2). Low (4°C) and high leaf temperatures (38–40°C) in light decrease Fv/Fm values and photosynthetic oxygen evolution at saturating CO_2_ (Streb et al., 2003a; Laureau et al., 2011) and *A*_*N*_ at ambient CO_2_ in *S. alpina* leaves (Streb, unpublished). Hence, after snowmelt, absorbed light energy is often largely in excess of what is consumed by carbon assimilation, supporting ROS formation. If ROS are not efficiently scavenged, they can promote photooxidative damage to the photosystems or the whole cell, while controlled ROS formation may lead to acclimation and improve the antioxidative defence system (Krieger‐Liszkay et al., 2008; Scheibe & Dietz, 2012; Coudhury et al., 2017; Scheibe, 2019; Fernandez‐Marin, 2020). It is, therefore, intriguing to attribute the high antioxidative protection capacities and particularly the high ascorbate content of NEW *S. alpina* leaves (Streb et al., 1998, 2003b) (Figure 6A) to their leaf survival during snowmelt. However, it was also shown that ascorbate in *S. alpina* was mostly localized outside the chloroplasts in the vacuole (Streb et al., 1997; Bligny & Aubert, 2012). Leaf ascorbate contents, glutathione contents and catalase activity decreased drastically during winter in SNOW leaves, weakening the antioxidant scavenging capacity. Ascorbate and glutathione may either be oxidised under snow in darkness to prevent general cellular oxidation (Davey et al., 2000) or distributed to actively growing flower shoots and flowers under snow (Körner et al., 2019) (Figure 6B). The lower antioxidant scavenging capacity of SNOW leaves is obviously sufficient to cope with ROS formation in the chloroplast in cold and light. In accord, declining glutathione contents to 50% in *S. alpina* leaves did not enhance photoinhibition at low temperatures (Laureau et al., 2011).

The conditions change rapidly after snowmelt due to a rise in leaf temperature and induce a rapid increase of antioxidant defence within days. The stimulation of catalase activity was most important, probably because of the onset of photorespiration after snowmelt. Except for ascorbate and glutathione reductase, this increase was transitioned probably because OLD leaves are successively shaded. Interestingly, all measured contents and activities of antioxidants and antioxidative enzymes were higher in senescent leaves compared to SNOW leaves. Enhanced ROS formation is thought to induce senescence (Krieger‐Liszkay et al., 2019). A weakening of antioxidative defence cannot be responsible for the induction of senescence of *S. alpina* leaves.

Carotenoids can scavenge singlet oxygen in PSII (Krieger‐Liszkay et al., 2000). The carotenoid content varied during the leaf life cycle similarly to the total chlorophyll content, keeping the chlorophyll/carotenoid ratio approximately constant. Only in senescent leaves does the chlorophyll *a* + *b* content decrease more pronounced than the carotenoid content. Both carotenoid and total chlorophyll content were similar in NEW and OLD leaves and slightly lower in SNOW leaves. The prolonged dark period in winter did not induce a major degradation of photosynthetic pigments. However, their new synthesis was markedly but transiently induced directly after snowmelt, which is similar to antioxidative protection. This was also described by Fernandez‐Marin (2021) in *S. alpina* collected at some distance from the snow.

A lower chlorophyll *a* + *b* content, chlorophyll *a*/*b* ratio, Fv/Fm ratio, *A*_*N*_, *J*_t_* and the 77 K fluorescence emission in SNOW compared to NEW leaves indicate downregulation of PSII. NEW leaves are mostly formed under shaded conditions, although mean daily PFDs may be as high as 800 μmol m^−2^ s^−1^ (Talhouët et al., 2019). Before snowfall, PFDs increase because the shading vegetation gets senescent. After the artificial sudden shade‐light transfer of *S. alpina* leaves at the growing site, photosynthesis declines, indicating downregulation or inactivation of photosynthesis (Talhouët et al., 2019). It is, therefore, possible that PSII was already downregulated before snowfall.

While pigment contents in OLD leaves return to values of NEW leaves several weeks after snowmelt, the 77 K fluorescence spectra indicate a shift of absorbed light energy in favour of PSI in OLD leaves. In accord, the oxidation state of Q_A_ (ql, Figure 3B) was higher in OLD compared to NEW leaves at PFDs higher than 750 μmol m^−2^ s^−1^. Furthermore, a slightly higher *J*_t_* in OLD compared to NEW leaves (Tables 4, S3) appears to support a higher ETRO in OLD leaves. Since photorespiration requires additional ATP compared to carbon assimilation (Cornic & Baker, 2012), this ATP may be generated by cyclic electron transport around PSI, as described in *Geum montanum*, another alpine snow‐bed species (Manuel et al., 1999). It should also be noted that NPQ at high PFD was highest in OLD leaves, indicating a higher proton gradient.

Although *A*_*N*_ was substantial in SNOW leaves, it was markedly lower than in NEW and OLD leaves. The *A*_*N*_ */C*_*I*_ curve of SNOW leaves compared to NEW and OLD resembled those of transgenic tobacco with reduced Rubisco activity (von Caemmerer, 2000). *S. alpina* leaves are thick, with up to three layers of palisade parenchyma cells and visibly little contact with internal air space (Talhouët et al., 2020). Such anatomical restraints suggest low conductivity to mesophyll diffusion of CO_2_, as shown for Antarctic plants (Saez et al., 2017). In accordance with this, the highest applied *C*_*I*_ was insufficient to saturate *A*_*N*_ in all three leaf types.

When absorbed PFD exceeds the activity of carbon assimilation and photorespiration, i.e. when both are saturated at 500 μmol m^−2^ s^−1^ PFD for SNOW and at 1000 μmol m^−2^ s^−1^ PFD for NEW and OLD leaves, one would expect that electron transport to alternative acceptors increases (Miyake 2010). This was not the case, as previously observed in *R. glacialis* leaves (Laureau et al. 2015). ETR_alt_ increased to a maximum at light saturation in all leaf types and then remained stable in SNOW leaves and decreased in NEW and OLD leaves. Alternative electron transport to oxygen as final electron acceptor other than photorespiration can protect PSII from photoinhibition by modulating the redox state of plastoquinone (Cornic & Baker, 2012). The nature and the relative importance of alternative electron pathways to oxygen in plants are controversial, and there exists evidence for the water–water‐cycle, the activity of PTOX and the malate valve (Laisk et al., 2007; Miyake, 2010; Ivanov et al., 2012; Cornic & Baker, 2012; Scheibe, 2019). Since *S. alpina* leaves have high ROS scavenging activity of water–water‐cycle enzymes and contents of antioxidative metabolites (Streb et al., 1998) but also high PTOX contents (Laureau et al., 2011), different pathways could contribute to keeping ROS formation under control. According to our calculation, ETR_alt_ accounted for 12% of electrons transported from PSII in SNOW leaves and up to 18% in NEW leaves. ETR_alt_ was, therefore, lower than ETRO (between 25 and 27%) and much lower than ETRC but still significantly high and closer to calculations of the water–water‐cycle reviewed by Miyake (2012) than to calculations of Laisk et al. (2007). The observation that alternative electron flow was lowest in SNOW leaves was surprising since ETRC and ETRO were also lowest.

Here, we characterised for the first time the photosynthetic behaviour and antioxidative protection of leaves, which are covered for 6 to 9 months in darkness under snow but are viable for at least two vegetation periods. While NEW and OLD leaves varied slightly in individual antioxidants and enzymes, they were downregulated in SNOW leaves, which was similar to photosynthetic activity. However, after snow‐melt leaves rapidly adjust antioxidant scavenging and shift light absorption from PSII to PSI. Electron transport to alternative acceptors potentially generating reactive oxygen did not increase in general with light intensity but was highest at light saturation and lower in SNOW leaves than in the other leaf types.

## AUTHOR CONTRIBUTIONS

PS designed the experiments, wrote the manuscript and did most of the experiments, PD and KS measured and analysed leaf absorption, GK added some experience in photosynthetic measurements and analysis of the models, GV performed the 77 k fluorescence spectra and added in redaction of the manuscript.

## Supporting information


**Data S1:** Thermographic photos showing temperature variation of *S. alpina* leaves during and after snowmelt.
**S2:** A list of abbreviations.
**S3:** Comparison of electron transport calculation with three different methods.
**S4:** Justification for polynomial electron transport calculation and potential negative electron transport.
**S5:** Statistic analysis of Figures 6, 7, 8.

## Data Availability

Data are available on request.
