# Supplementary material for: Estimation of light utilisation and antioxidative protection in an alpine plant species (Soldanella alpina L.) during the leaf life cycle at high elevation
Source: Physiol Plant. 2025 Jan 16;177(1):e70045. doi: 10.1111/ppl.70045 (PMC11738846; doi:10.1111/ppl.70045)
Supplement: Supplementary file 1 — Data S1: Thermographic photos showing temperature variation of S. alpina leaves during and after snowmelt. S2: A list of abbreviations. S3: Comparison of electron transport calculation with three different methods. S4: Justification for polynomial electron transport calculation and potential negative electron transport. S5: Statistic analysis of Figures 6, 7, 8. [file PPL-177-e70045-s001.pdf]

Estimation of light utilisation and antioxidative protection in an alpine plant species (*Soldanella alpina* L.) during the leaf life cycle at high elevation

Peter Streb[1], Philippine Dubertrand[1], Gabriel Cornic[1], Kamel Soudani[1], Giovanni Finazzi[2]

Supporting information S1
Temperature conditions of *S. alpina* leaves after snowmelt

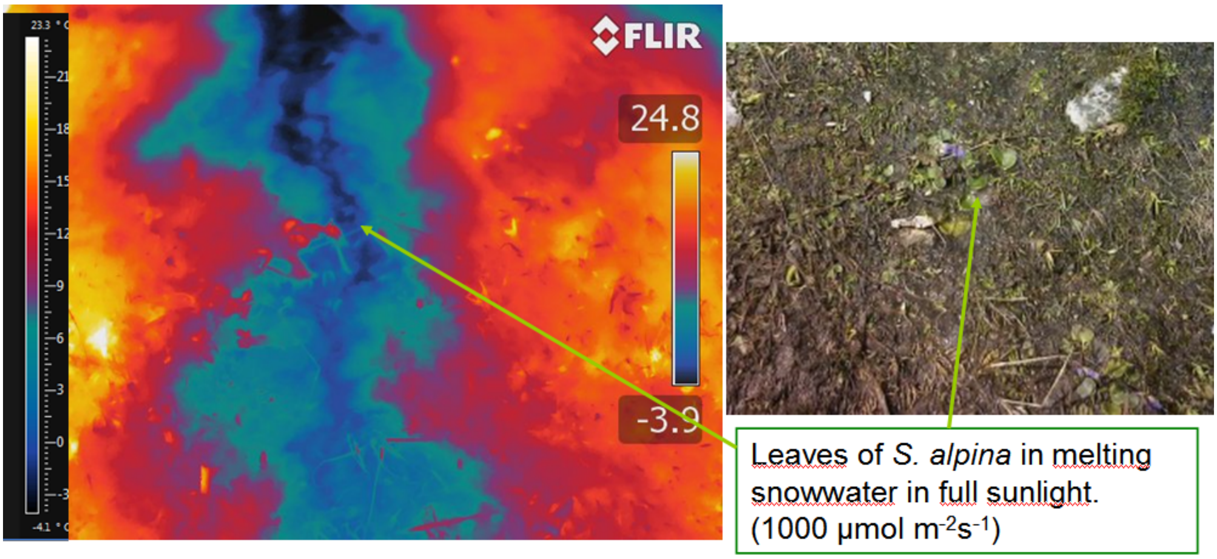

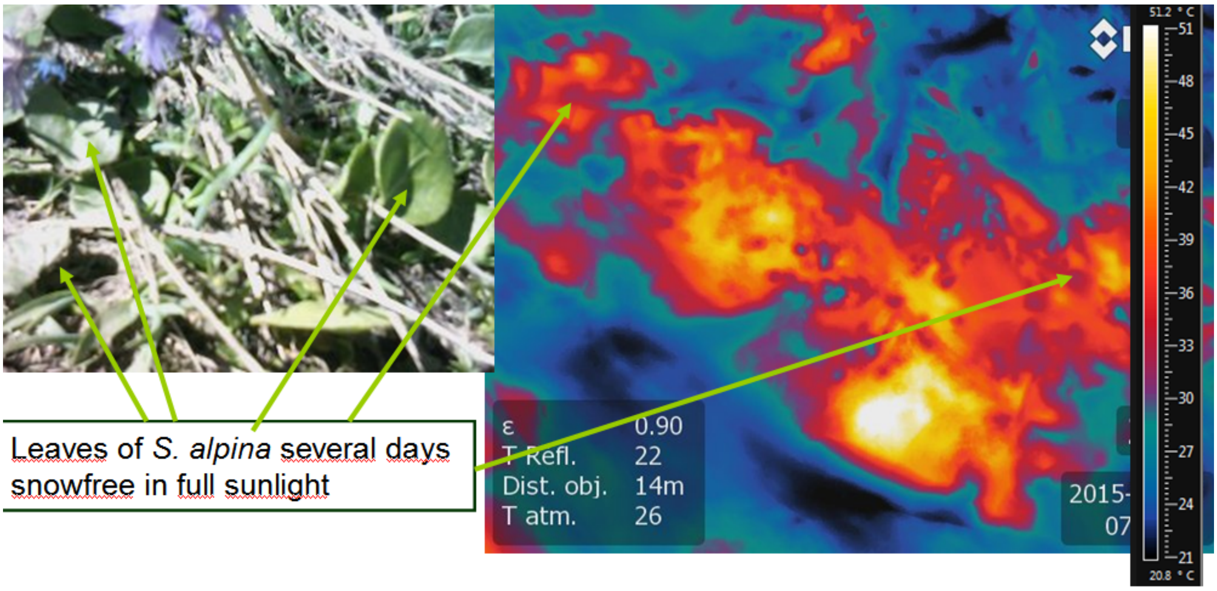

Leaves of *S. alpina* in full sunlight, either in melting snow (leaf temperature around 0°C) or several days later snowfree (leaf temperature 40°C)

Estimation of light utilisation and antioxidative protection in an alpine plant species (*Soldanella alpina* L.) during the leaf life cycle at high elevation

Peter Streb[1], Philippine Dubertrand[1], Gabriel Cornic[1], Kamel Soudani[1], Giovanni Finazzi[2]

Supporting information S2
Abbreviations used in the manuscript

A_N	carbon net assimilation
APx	ascorbate peroxidise
C^*	intercellular CO_2 compensation point in the absence of mitochondrial respiration
C_A	CO_2 partial pressure outside the leaf
C_I	CO_2 partial pressure inside the leaf
ETR_{alt}	alternative electron flow
ETRC	electron flow to carboxylation of RUBISCO
ETRO	electron flow to oxygenation of RUBISCO
Fm	maximum chlorophyll fluorescence after dark acclimation
Fm`	maximum chlorophyll fluorescence in light
Fo	minimum chlorophyll fluorescence after dark acclimation
Fo`	minimum chlorophyll fluorescence in light after far red illumination
Fv	variable chlorophyll fluorescence (Fm-Fo)
Ft	Fluorescence in light
Γ^*	chloroplastic CO_2 compensation point in the absence of mitochondrial respiration
gs	stomatal conductance
J_A	electron transport to carboxylation and oxygenation as calculated according to von Caemmerer (2000)
J_t	Calculated electron transport according to Krall & Edwards (1992)
J_t^*	Calculated electron transport as described in Material and Methods
NPQ	non-photochemical fluorescence quenching
PFD	photon flux density µmol $m^{-2}s^{-1}$
PFD_{abs}	absorbed photon flux density µmol $m^{-2}s^{-1}$
ϕPSII	PSII operating efficiency
ql	relative reduction state of PSII
R_L	respiration in light
R_N	dark respiration
ROS	reactive oxygen species
S_{appci}	apparent specificity factor of Rubisco (C_I – dependent)
V_c	carboxylation velocity of Rubisco
V_o	oxygenation velocity of Rubisco

Estimation of light utilisation and antioxidative protection in an alpine plant species (*Soldanella alpina* L.) during the leaf life cycle at high elevation

Peter Streb[1], Philippine Dubertrand[1], Gabriel Cornic[1], Kamel Soudani[1], Giovanni Finazzi[2]

Supporting information S3
Comparison of electron transport calculated by 3 different methods

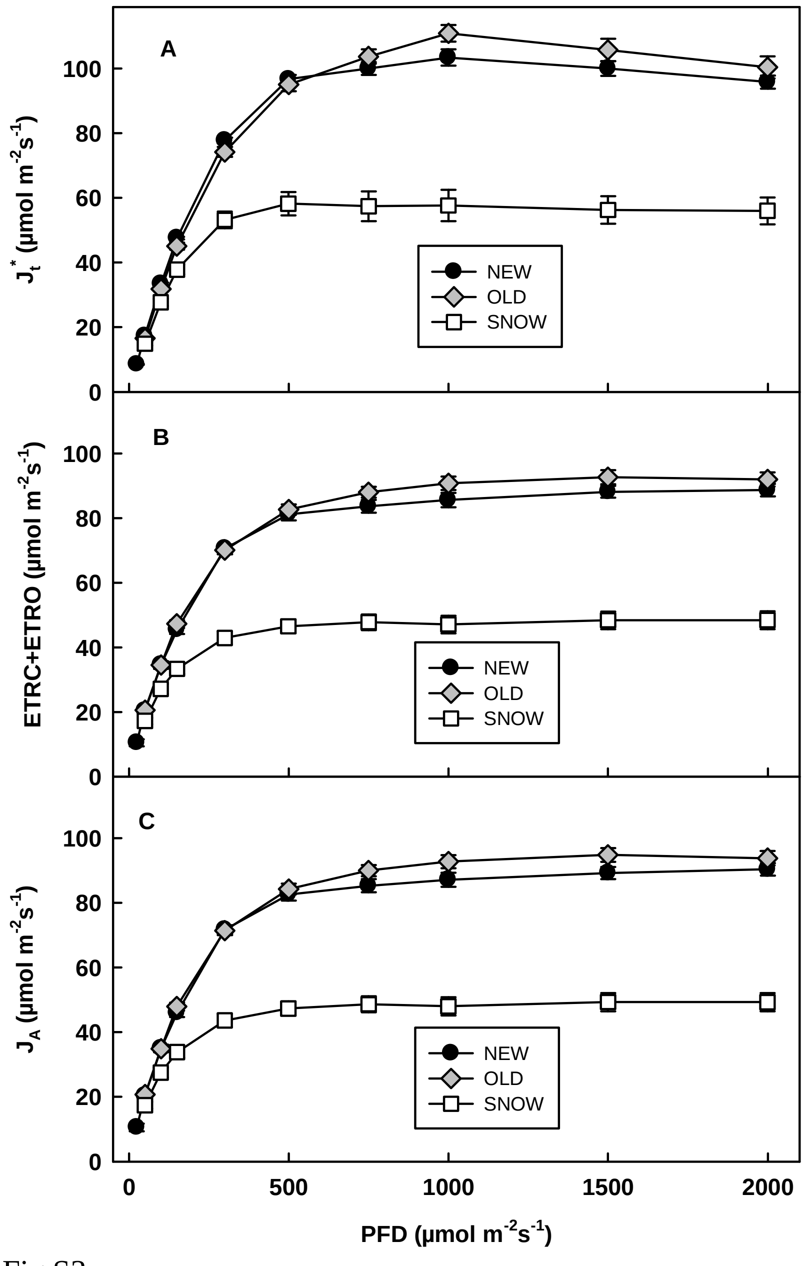

Fig.S3
Electron transport rates of S. alpina NEW (n ≥ 9), SNOW (n ≥ 5) and OLD (n ≥ 7) leaves. Total electron transport (J_t^*) was recalculated from fluorescence measurements, electron transport to carboxylation and oxygenation was calculated as described in Material and Methods (ETRC+ETRO) using S_{appCI} for OLD leaves and calculated day respiration according to Laisk. J_A was calculated according to von Caemmerer (2000), using C^* and day respiration

Estimation of light utilisation and antioxidative protection in an alpine plant species (*Soldanella alpina* L.) during the leaf life cycle at high elevation

Peter Streb[1], Philippine Dubertrand[1], Gabriel Cornic[1], Kamel Soudani[1], Giovanni Finazzi[2]

Supporting information S4
Calculation of PSII electron transport

S. alpina leaves are thick containing up to three layers of palisade parenchyme cells (Talhouët et al. 2020). Chlorophyll fluorescence is emitted by the upper cells, whereas gas exchange measures the mean of all chloroplasts participating in photosynthesis. In order to correlate electron transport, as measured by chlorophyll fluorescence, with gas exchange two problems are obvious (Evans 2009)

1) A light gradient within the leaves with higher light intensity at the uppermost chloroplasts. Consequently, electron transport rates calculated from chlorophyll fluorescence can be expected to be lower than the mean of all chloroplasts.

2) A CO_2 gradient, with higher CO_2 partial pressure at the lowermost chloroplast near the stomates. This can be expected to result in a gradient of ϕCO_2, since uppermost chloroplasts would suffer from a lower CO_2/O_2 ratio, leading to enhanced photorespiration.

It can therefore be expected that calculating PSII electron transport with the widely used calculation (Krall & Edwards 1992) ($Jt = 0.5 \times \phi\text{PSII} \times \text{PFD} \times \text{Abs}$) does not reflect PSII electron transport in all parts of the investigated leaf section. Previously, this problem was solved by plotting ϕCO_2 against ϕPSII in the absence of oxygen, suggesting that electrons can only be used for carbon assimilation, including minor reactions like nitrogen assimilation. Often a linear correlation between both parameters was measured in several plant species (Genty et al. 1990, Ghashghaie & Cornic 1994, Streb et al. 2005, Laureau et al. 2013). This linear correlation was subsequently applied to calculate a hypothetical ϕCO_2^* using ϕPSII under atmospheric conditions, assuming that the relation between ϕCO_2 and ϕPSII is the same in the absence and the presence of oxygen (Laureau et al. 2013). The hypothetic ϕCO_2^* was used to recalculate the electron transport at PSII as $J_t^* = \phi CO_2^* \times 4 \times \text{Abs}$. Often recalculated Jt^* is slightly lower than electron transport as estimated by Krall & Edwards (1992) (Laureau et al. 2013). In contrast, the relation between ϕCO_2 and ϕPSII in *S. alpina* leaves is not strictly linear (Fig. S4a). We tested therefore, in addition, a polynomial and a sigmoid correlation between ϕCO_2 and ϕPSII in all leaf types and separately for A_N/light and A_N/Ci curves.

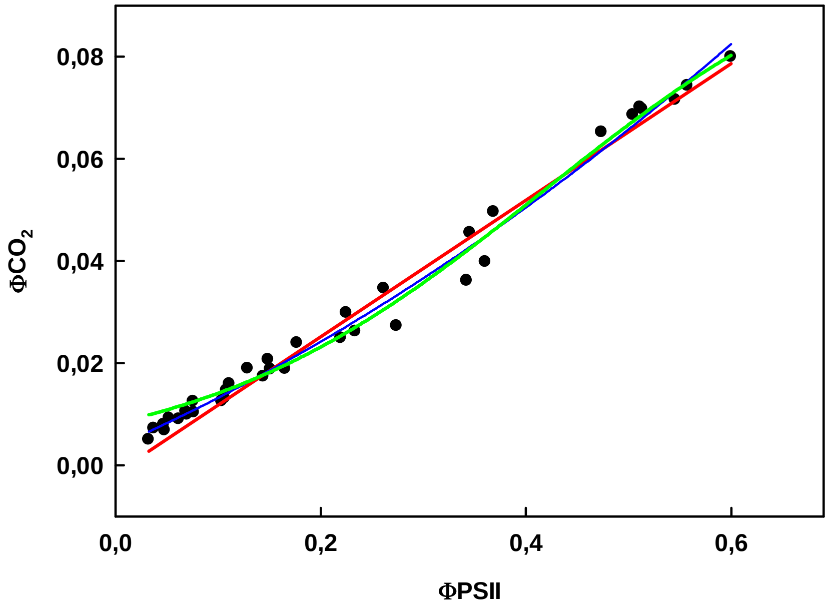

Fig. S4a
Relation between ϕPSII and ϕCO_2 in NEW *S. alpina* leaves measured in the absence of oxygen from A_N/light curves. The linear correlation is shown in red, the polynomial correlation in blue and the sigmoid correlation in green.

In order to verify the best correlation, we recalculated Jt^* in the absence of oxygen, suggesting that the recalculated mean Jt^* should match the mean A_N (Jt^*-A_N =0). Results are shown in Fig. S4b and compared to the Krall & Edwards (1992) calculation of *Jt*. The smallest deviation from the expected zero (4 e^- µmol $m^{-2}s^{-1}$) over all PFDs was obtained by a recalculation of Jt^* with a polynomial regression which was thereafter used to recalculate Jt^* under atmospheric conditions. The same result was obtained for all leaf types under all measured conditions.

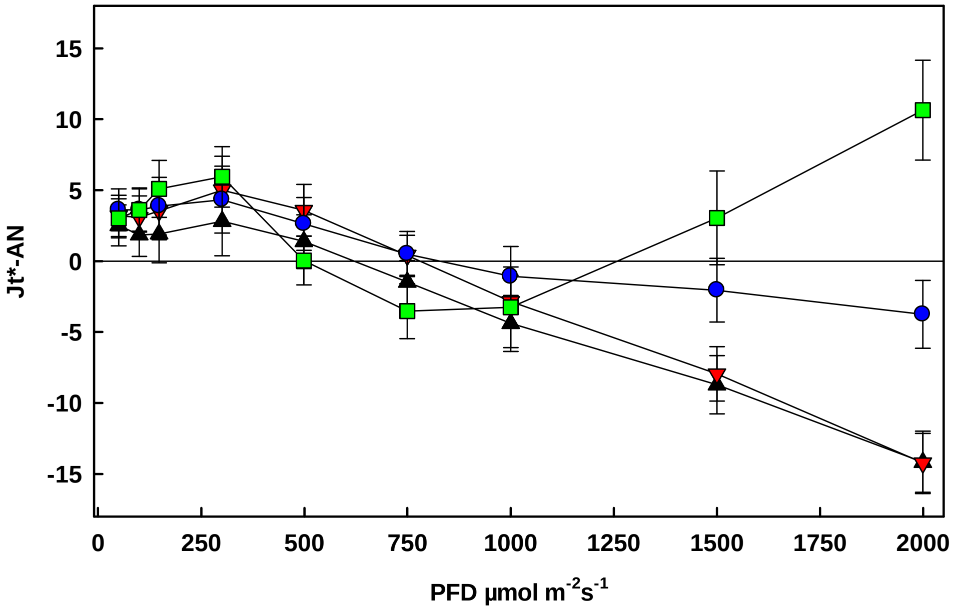

Fig. S4b

A_N in the absence of oxygen was subtracted from total electron transport under the same conditions. Total electron transport was either calculated according to Krall & Edwards (1992) (black) or recalculated after linear calibration (red), polynomial calibration (blue) or sigmoid calibration (green)

At low PFD, a negative value was calculated for ETR_{alt} (Fig. 4C) corresponding to up to 4 e^- µmol $m^{-2}s^{-1}$. This difference may either indicate the limit of the model or can be explained by another effect. When calculating the percentage of ETRO relative to the total *Jt** (not shown), ETRO was highest at PFDs which results in a negative ETR_{alt} for all leaf types. At higher PFD, ETRO in % of total *Jt** was nearly constant. Low PFDs may be absorbed by the uppermost chloroplast only. These chloroplasts can be expected to have the lowest CO_2/O_2 ratio under atmospheric conditions, favouring photorespiration. However, the calibration between ϕCO_2 and ϕPSII was done in the absence of photorespiration and therefore the correlation was not precise at low PFD under atmospheric conditions.

Estimation of light utilisation and antioxidative protection in an alpine plant species (*Soldanella alpina* L.) during the leaf life cycle at high elevation

Peter Streb[1], Philippine Dubertrand[1], Gabriel Cornic[1], Kamel Soudani[1], Giovanni Finazzi[2]

Supporting information S5

Statistic analysis of Figs. 6 -8 of the manuscript.
All analysis were done using the T-Test of the Sigma plot programme comparing pare wise all data. Statistic difference was suggested to be significant at the p<0.05 level and are indicated as Y. Non-statistic difference is indicated by N.

Fig. 6A Ascorbate content

		NEW End season	SNOW	SNOW +3 days	SNOW +10 days	OLD	<50% senescent	>50% senescent
NEW	Start season	N	Y	Y	Y	Y	Y	Y
NEW	End season		Y	Y	Y	Y	Y	Y
SNOW				Y	Y	Y	Y	N
SNOW	+3 days				N	Y	N	Y
SNOW	+10 days					N	N	Y
Old							N	Y
<50%	senescent							N

Fig. 7A GSH content

	SNOW	OLD
NEW	Y	N
SNOW		Y

Fig. 7B glutathione reductase activity

		SNOW	SNOW +3 days	SNOW +10 days	OLD	Senescent
NEW		N	Y	Y	N	N
SNOW			Y	Y	Y	N
SNOW	+3 days			N	N	N
SNOW	+10 days				N	Y
OLD						N

Fig. 7C Catalase activity

		SNOW	SNOW +3 days	SNOW +10 days	OLD	Senescent
NEW		Y	N	Y	N	N
SNOW			Y	Y	Y	Y
SNOW	+3 days			Y	N	N
SNOW	+10 days				Y	Y
OLD						N

Fig. 7D Ascorbate peroxidase activity

		SNOW	SNOW +3 days	SNOW +10 days	OLD	Senescent
NEW		N	Y	N	N	N
SNOW			Y	N	N	N
SNOW	+3 days			N	Y	N
SNOW	+10 days				N	N
OLD						N

Fig. 8A Chlorophyll a+b content

		SNOW	SNOW +3 days	SNOW +10 days	OLD	Senescent
NEW		N	N	Y	N	Y
SNOW			Y	Y	Y	Y
SNOW	+3 days			N	N	Y
SNOW	+10 days				Y	Y
OLD						Y

Fig. 8B Carotenoid content

		SNOW	SNOW +3 days	SNOW +10 days	OLD	Senescent
NEW		N	N	N	N	Y
SNOW			Y	Y	N	N
SNOW	+3 days			N	N	Y
SNOW	+10 days				Y	Y
OLD						Y

Fig. 8C Chlorophyll a/b ratio

		SNOW	SNOW +3 days	SNOW +10 days	OLD	Senescent
NEW		Y	Y	Y	N	N
SNOW			Y	Y	Y	N
SNOW	+3 days			Y	Y	Y
SNOW	+10 days				Y	Y
OLD						N
